# Case Report: Sequential lymphomas: may HLA system play a role in this uncommon phenomenon?

**DOI:** 10.3389/fonc.2025.1586454

**Published:** 2025-06-24

**Authors:** Evgenia Verrou, Ioanna Diamanti, Asimina Fylaktou, Aikaterini Daiou, Dimitra Dalampira, Xanthippi Dimitriadou, Vassiliki Tara, Elissavet Karadrakonti, Theodora Triantafyllou, Aggeliki Sevastoudi, Efthimia Vlachaki, Nikolaos Karampatzakis, Theodosia Papadopoulou, Emmanouil Sinakos, Eirini Katodritou, Georgia Gioula

**Affiliations:** ^1^ Hematology Department, Theagenio Cancer Hospital, Thessaloniki, Greece; ^2^ Biochemistry and Microbiology Department, Theagenio Cancer Hospital, Thessaloniki, Greece; ^3^ National Peripheral Histocompatibility Center-Immunology Department, Hippokration General Hospital, Thessaloniki, Greece; ^4^ 2nd Department of Internal Medicine, Hippokration General Hospital, School of Medicine, Faculty of Health Sciences, Aristotle University of Thessaloniki, Thessaloniki, Greece; ^5^ 4th Department of Internal Medicine, Hippokration General Hospital, School of Medicine, Faculty of Health Sciences, Aristotle University of Thessaloniki, Thessaloniki, Greece; ^6^ Microbiology Department, School of Medicine, Faculty of Health Sciences, Aristotle University of Thessaloniki, Thessaloniki, Greece

**Keywords:** sequential lymphomas, DLBCL, cHL, HLA system, autoimmunity

## Abstract

The sequential occurrence of diffuse large B‐cell lymphoma (DLBCL) in a patient diagnosed with classical Hodgkin lymphoma (cHL) or vice versa represents a rare situation. In parallel, human leukocyte antigen (HLA) has been studied extensively about rising susceptibility in various lymphomas. Herein, we present clinical characteristics, the outcome and the results of HLA class-I and class-II investigation in patients sequentially diagnosed with the above-mentioned combination of lymphomas. We describe 8 patients (6 males/2 females) with median age at diagnosis of first and second lymphomas of 45.5 years (range: 25–74 years) and 57.5 years (range: 30–83 years), respectively. The median interval between the first and the second diagnosis was 6.5 years (range: 4–22 years). Regarding HLA investigation, we observed that four of our patients were HLA‐DQB1*03:01 positive. Interestingly, three of our patients displaying this HLA allele developed a third lymphoma. Notably, we observed that the HLA profile of three other patients revealed the presence of HLA-B*35:03. Interestingly, both above-mentioned HLA alleles have been associated with autoimmune manifestations. Although the presence of certain HLA alleles in our patients could be coincidental, our results suggest that HLA typing may be a field of investigational interest regarding patients with sequential lymphomas.

## Introduction

The metachronous development of different histologic types of lymphoma in a single patient has been previously described as sequential lymphomas (1). Classical Hodgkin lymphoma (cHL) is a lymphoid neoplasm of B-cell origin, although the sequential occurrence of B-cell non-Hodgkin Lymphoma (NHL) in a patient diagnosed with cHL is an uncommon situation (2, 3). More specifically, case series of sequential lymphomas involving both cHL and primary mediastinal B‐cell lymphoma (PMBCL) have been sporadically reported (4, 5). In contrast, initial diagnosis of cHL followed by a distinct diagnosis of diffuse large B‐cell lymphoma (DLBCL) or vice versa is a rare phenomenon (3, 6). As a result, management and outcome information of these patients are poorly covered in the literature. Additionally, a biological interest is raised by these cases, as it remains unclear whether the occurrence of sequential lymphomas is attributed to plasticity of malignant clone or to susceptibility for developing lymphomas.

Multiple etiological factors are involved in lymphoma pathogenesis (7). In parallel, the major histocompatibility complex (MHC) has been studied extensively about rising susceptibility in various autoimmune diseases, as well as malignant neoplasms. Interestingly, human leukocyte antigen (HLA) homozygosity has been associated with cancer susceptibility (8). Regarding lymphomas, it has been hypothesized that the participation of HLA in tumor antigen presentation could also be involved not only in pathogenesis, but in disease control as well (9–12). More specifically, structural or functional alterations of the HLA molecule on the surface of malignant cells may inhibit presentation of the tumoral antigen to cytotoxic T cells, suggesting that neoplastic clone may be undetectable by T cells. Moreover, mutations leading to the aberrant cytoplasmic localization of the HLA molecule may also influence antigen presentation.

Focusing on DLBCL, HLA-class I loss is more commonly observed than HLA-class II loss (13). Furthermore, in a study conducted by Alcoceba et al., comparing 250 DLBCL cases to 1940 controls of European origin it was shown that the phenotypic frequency of HLA-DRB1*01 was statistically significantly higher in DLBCL patients than in the control group (14). Additionally, Wang et al., compared 610 NHL cases and 555 controls of non-Hispanic white descent in the US, indicating that HLA-A*26:01 and HLA-B*51:01 may increase the risk for developing DLBCL (15).

Regarding cHL, several studies have shown that HLA‐A*01:01 and HLA-A*02:01 are associated with an increased risk or decreased risk of Epstein Barr Virous (EBV)‐positive cHL, respectively (16, 17). Moreover, a recently published study showed protective effects of heterozygosity regarding HLA-class I and HLA-class II locus for cHL (18).

Due to the rarity of sequential lymphomas, it has not been investigated whether, HLA diversity is associated with increased susceptibility for developing different histological types of lymphomas in the same patient.

## Methods

To gain insight into the lymphomagenesis of sequential lymphomas, we retrospectively recorded the medical history, clinical characteristics and the outcome of patients with sequential diagnosis of cHL and DLBCL or vice versa treated in our Center between 1990 and 2023. Out of eight patients included in the study, four patients had a reviewed histopathology of the lymphoma subtype according to WHO 2022 classification. For the rest of the patients, diagnoses were based on the histopathology reports included in the patient’ s records. Additionally, considering the significance of HLA-related susceptibility for lymphomas, we aimed to present HLA class I (HLA‐A, HLA‐B, HLA‐C) and HLA class II (HLA‐DRB1, HLA‐DQB1) in patients diagnosed with this combination of lymphomas. HLA typing for HLA‐A*, -B*, -C*, -DRB1* and -DQB1*was performed using Sequence‐Specific Oligonucleotide probes (SSO) and Sequence‐Specific Primer (SSP).

## Results

Our study included eight patients of Greek origin with sequential diagnosis of cHL and DLBCL-NOS, two females (cases 3 and 4) and six males. Regarding medical history, all patients were negative for HIV, HBV and HCV infection, and only one patient mentioned a medical history of autoimmune disease (case 2). None of the patients revealed any occupational exposure to substances related to lymphomagenesis. The first diagnosis was cHL in six patients while two patients were initially diagnosed with DLBCL and sequentially developed cHL (cases 4 and 5). The median age at diagnosis of all the first and second lymphomas was 45.5 years (range: 25–74 years) and 57.5 years (range: 30–83 years), respectively. Five patients developed the second lymphoma within 10 years of the diagnosis of the first lymphoma. However, three patients (cases 1, 4, and 8) displayed lymphadenopathy more than 10 years after their initial disease, resulting in diagnosis of the second lymphoma. Consequently, the median interval between the first and the second lymphoma was 6.5 years (range: 4–22 years).

Importantly, five patients have been alive at the time of the study and in remission for both lymphomas. In contrast, three patients (37.5%) died due to hematologic disease resulting in a 5-year and 10-year overall survival after the diagnosis of the second lymphoma of 85.7% and 51.4%, respectively. Interestingly, all three above-mentioned patients experienced a relapse of the first or second diagnosed lymphoma and, additionally, they developed a third lymphoproliferative neoplasm. More specifically, one male patient (case 2) was initially diagnosed with cHL and six years later he developed DLBCL and was treated with Rituximab- Cyclophosphamide-Doxorubicin-Vincristine-Prednisone (RCHOP) reaching complete response (CR). Two years later, he experienced relapse of DLBCL and received four cycles of Rituximab-Dexamethasone-High dose AraC-Cis-platinum (RDHAP) as autologous transplantation was not possible, due to inadequate mobilization of stem cells. Three years later, he developed nodular lymphocyte-predominant HL (NLPHL) and received Rituximab- Cyclophosphamide-Vincristine-Prednisone (RCVP) achieving CR. While being in remission for all lymphomas, he displayed persistent pancytopenia and died due to sepsis. Normal karyotype [46, XY] was documented by cytogenetic analysis, while NGS revealed normal results regarding myelodysplasia-related genes. Bone marrow aspirate and biopsy showed reduced cellularity and mild myelodysplasia of all lines. A second male patients (case 5) developed sequentially DLBCL, cHL and NLPHL. He was treated successfully for all types of lymphomas, but experienced relapse of DLBCL and died due to disease refractoriness. Finally, a female patient (case 4) was treated with CHOP for DLBCL and developed cHL 21 years after treatment for DLBCL. Due to cardiovascular comorbidities, she was treated with brentuximab vedotin as monotherapy for cHL and, after achieving CR, she developed mycosis fungoides requiring only local therapy. Finally, she experienced a relapse of cHL but denied further treatment due to advanced age. Clinicopathological characteristics and outcomes of the patients displaying sequential lymphomas are summarized in **Table 1**, while staging and treatment modalities of cHL and DLBCL are included in **Table 2**.

**Table 1:** Clinical features and outcome of patients with sequential lymphomas.

Case /Sex	Diagnoses	Age at 1st lymphoma (years)	Interval between diagnoses (years)	Re-occurrence of 1^st^ or 2^nd^ lymphoma	Third Lymphoma	OS after 2^nd^ Lymphoma (years)	Status
#1/M	cHL/DLBCL	50	22	Yes	–	12	Alive
#2/M	cHL/DLBCL	41	6	Yes	NLPHL	9	Dead
#3/F	cHL/DLBCL	74	4	No	–	5	Alive
#4/F	DLBCL/cHL	64	21	Yes	Mycosis Fungoides	3	Dead
#5/M	DLBCL/cHL	61	7	Yes	NLPHL	7	Dead
#6/M	cHL/DLBCL	25	5	No	–	17	Alive
#7/M	cHL/DLBCL	40	4	No	–	2	Alive
#8/M	cHL/DLBCL	31	16	No	–	21	Alive

cHL, classical Hodgkin lymphoma; DLBCL, diffuse large B‐Cell lymphoma; NLPHL, nodular lymphocyte-predominant HL.

**Table 2:** Staging and treatment modalities of sequential lymphomas.

Case /Sex	1st Diagnosis	Stage	Treatment	2nd Diagnosis	Stage	Treatment	Treatment of Relapse
#1/M	cHL	3	ABVD	DLBCL	4	RCHOP	R-DHAP
#2/M	cHL	3	ABVD	DLBCL	4	RCHOP	R-DHAP
#3/F	cHL	2	ABVD+RT	DLBCL	4	RCHOP	NA
#4/F	DLBCL	4	CHOP	cHL	4	Brentuximab -Vedotin	Denied
#5/M	DLBCL	3	RCHOP	cHL	3	ABVD	Gemcitabine-Vinorelbine
#6/M	cHL	3	ABVD	DLBCL	3	RCHOP	NA
#7/M	cHL	3	ABVD	DLBCL	4	RCHOP	NA
#8/M	cHL	3	ABVD	DLBCL	3	RCHOP	NA

cHL, classical Hodgkin lymphoma; DLBCL, diffuse large B‐Cell lymphoma; ABVD, Adriamycin- Bleomycin -Vinblastine- Dacarbazine; RCHOP; Rituximab- Cyclophosphamide -Doxorubicin- Vincristine-Prednisone; R-DHAP, Rituximab-Dexamethasone-High dose AraC-Cis-platinum; RT, Radiotherapy; NA, Not Applicable.

Regarding HLA investigation, we observed that four of our patients were HLA‐DQB1*03:01 (DQ7) positive (cases 2,3,4, and 5). Three of our patients displaying this HLA allele were diagnosed with a third lymphoma (cases 2,4, and 5). Moreover, all three patients experienced also relapses of DLBCL or cHL resulting in an unfavorable prognosis. Additionally, we observed that the HLA profile of three patients revealed the presence of HLA-B*35:03 (cases 4, 6, and 7). Both male patients (cases 6 and 7) were initially diagnosed with cHL and developed sequentially DLBCL within 5 years from the first diagnosis and have been alive and in remission for both lymphomas at the time of the study. HLA typing of patients developing sequential lymphomas is summarized in **Table 3**.

**Table 3:** HLA results of patients with sequential lymphomas.

Case/Sex	HLA-A*		HLA-B*		HLA-C*		HLA-DRB1*		HLA-DQB1*	
1#/M	03	68	44	51	02	15	14	15	05:03	06:02
2#/M	02	02	18	40	02	07	11	16	03:01(DQ7)	05:02
3#/F	24	32	35:02	51:01	04	14	11	11	03:01(DQ7)	03:01(DQ7)
4#/F	02	02	18:01	35:03	04	07	11	11	03:01(DQ7)	03:01(DQ7)
5#/M	23	33	14	49	07	08	01	04	03:01(DQ7)	05:01
6#/M	02	02	35:03	51:01	04	15	16	16	05:02	05:02
7#/M	01	01	35:03	37:01	04	06	13	14	05:03	06:04
8#/M	01	11	08	57	06	07	03	16	02:01	05:02

HLA, human leukocyte antigen.

## Discussion

Both cHL and DLBCL have been extensively investigated as separate entities, but they are poorly studied in the literature as metachronous pathologies. In parallel, although HLA has been widely studied in respect to patients’ susceptibility for lymphomas, to our knowledge, this is the first report of HLA investigation in patients with sequential lymphomas. HLA‐ DQB1*03:01(DQ7) was observed in four patients, while three of them developed a third lymphoproliferative disease and one of them had also a history of Adamantiadis-Behcet’s disease. Importantly, HLA‐DQB1*03:01(DQ7) has been previously reported among patients with autoimmune dermatological manifestations. More specifically, literature demonstrates that HLA‐DQB1*03:01(DQ7) is the most frequently observed HLA-Class II allele in patients representing all the clinical variants or subsets of pemphigoid disease, in a statistically significant correlation (19). However, it is well established that autoimmune disorders can be associated with increased future risk of lymphoproliferative malignancies (7, 20). Therefore, it remains to be clarified whether HLA‐DQB1*03:01(DQ7) allele may be involved in antigen presentation resulting in a common base of autoimmunity and lymphomagenesis.

Additionally, three of our patients displayed the HLA-B*35:03 allele. Interestingly, HLA-B*35:03 has been also reported in patients who developed subacute thyroiditis (SAT-also called de Quervain’s disease) after vaccination against SARS-CoV-2 (21). Generally, SAT appears to be a rare side effect of vaccination. One of the proposed mechanisms of vaccine-induced SAT induction is an autoimmune/inflammatory syndrome (ASIA) evoked by adjuvants. It is suggested that ASIA occurs mainly in genetically predisposed people (22). It could be hypothesized, that overaction to external stimuli may also be involved in the pathobiology of sequential lymphomas. Undoubtfully, a major limitation of our study is that it includes a very small number of cases. Due to the very small number of patients, it was not made possible in the present study to proceed with a statistical analysis of our data, so that statistical correlations could be inferred. Therefore, any potential correlation between the presence of certain alleles observed in our study and the occurrence of sequential lymphomas cannot be suggested, although it cannot be rejected either. However, our results suggest that HLA genotyping may be an important variable for understanding the contributory mechanisms for the development of sequential lymphomas. To enhance the interpretation of our findings, we will continue collecting new data of sequential lymphomas cases, aiming to compare the HLA polymorphisms with those observed in patients displaying only one type of lymphoma.

Sequential lymphomas may be associated with the plasticity of neoplastic clone, with an immunological deficiency or both. Environmental and occupational factors are also associated with an increased risk of lymphomas (7). A possible etiological model might also include patients having a genetic predisposition to develop multiple lymphoproliferative diseases. Moreover, chemotherapy delivered for the first diagnosis may induce immunodeficiency or genetic alterations leading to the development of secondary lymphomas (23). Therefore, our cases underscore the complexity of lymphomas and the necessity to perform biopsies in every relapse of the initial disease.

Attarbaschi et al. recently described 189 children or adolescents initially diagnosed with NHL developing second malignant neoplasms (24). Lymphoid neoplasms were observed as second diagnosis in 51 patients (27%). Second lymphoid neoplasms included 11 patients with acute lymphoblastic leukemia, 9 with HL and 31 with NHL. The median time of occurrence for second lymphoid neoplasms was 4.38 years (0.80–20.21 years). Five-year OS after diagnosis of second lymphoma for the 51 patients was 59% while twenty-four patients (47%) died due to lymphoid neoplasms. These results are in accordance with our observations regarding the time of occurrence of second lymphoma in patients developing sequential lymphomas. However, our patients showed a more favorable prognosis compared to those reported in the study of Attarbaschi et al. This difference is likely to be explained by the fact that Attarbaschi et al., included in their study patients who also developed ALL as secondary lymphoid neoplasm.

Focusing on the time of occurrence of second lymphoma we observed a heterogeneity among our group of patients. Three patients displayed a very delayed diagnosis of second lymphoma, and this could be explained by the fact that lymphoma patients due to improvement of therapeutic interventions live long enough to develop a secondary malignancy. In these cases, genetic susceptibility for developing cancer could be more prominent. In contrast, five patients developed second lymphoma within 10 years of first malignancy and for those patients it could hypothesized that plasticity of neoplastic clone, environmental factors or therapy-related alterations could play an important role in the pathogenesis of the second neoplasia.

Recently Virga et al., described the occurrence of NHL as secondary malignancy in patients treated for cHL (3). In a cohort of 164 cHL patients, five patients were identified with DLBCL during lymphoma relapse. In one patient, IgHV gene rearrangements were investigated by PCR, and a consistent pattern was reported for both cHL and DLBCL samples. This finding supports a common origin and a clonal relationship between sequential lymphomas. A limitation of our study was the lack of data regarding possible common clonality of sequential lymphomas.

Interestingly, three of our patients developed a third lymphoid malignancy. The development of three distinct lymphoid malignancies in the same patient is an extremely rare manifestation. In these cases, the clonal relationship of the underlying diagnoses is also of great interest. Recently, Salvetti et al., described the sequential occurrence of three mature lymphoid neoplasms in a single patient showing a lack of clonal correlation between the diagnosed lymphomas (25). Authors suggested that the diagnosis of distinct lymphoid neoplasms might have been partially favored by extrinsic microenvironmental mechanisms, including impaired immunological surveillance. A similar clinical and biological scenario could be also suggested for our three patients who developed a third lymphoma displaying an unfavorable course leading to death due to lymphoma.

In conclusion, the occurrence of sequential lymphomas in our patients could be explained by multiple etiological factors. Therefore, we believe that our cases emphasize the necessity for collecting and investigating sequential lymphoma patients in order to understand the underlying contributory mechanisms in the pathogenesis of relapsed lymphomas. In parallel, our results suggest that HLA typing may be a field of investigational interest regarding patients with sequential lymphomas.

## Data Availability

The raw data supporting the conclusions of this article will be made available by the authors, without undue reservation.

## References

[1] Siebert JD Mulvaney DA Vukov AM Knost JA King DE Craig FE . Utility of flow cytometry in subtyping composite and sequential lymphoma. J Clin Lab Anal. (1999) 13:199–204. doi: 10.1002/(SICI)1098-2825(1999)13:5<199::AID-JCLA1>3.0.CO;2-W 10494126 PMC6807893

[2] Eichenauer DA Müller H Elger L Goergen H Fuchs M Kreissl S . Non-Hodgkin lymphoma after treatment for classical Hodgkin lymphoma: a report from the German Hodgkin Study Group. Br J Haematol. (2021) 193:515–9. doi: 10.1111/bjh.v193.3 33486762

[3] Virga B Pinczés L Illés Á Miltényi Z Magyari F Méhes G . Occurrence of secondary non-Hodgkin lymphomas among our classical Hodgkin lymphoma patients: A single-centre experience. Cureus. (2024). Available online at: https://www.cureus.com/articles/253727-occurrence-of-secondary-non-hodgkin-lymphomas-among-our-classical-hodgkin-lymphoma-patients-a-single-centre-experience. 10.7759/cureus.63307 PMC11283309 39070524

[4] Vassilakopoulos TP Piperidou A Hadjiharissi E Panteliadou AK Panitsas F Vassilopoulos I . Development of Classic Hodgkin Lymphoma after successful treatment of primary mediastinal large b-cell lymphoma: results from a well-defined database. Leuk Res. (2021) 100:106479. doi: 10.1016/j.leukres.2020.106479 33285316

[5] Aussedat G Traverse-Glehen A Stamatoullas A Molina T Safar V Laurent C . Composite and sequential lymphoma between classical Hodgkin lymphoma and primary mediastinal lymphoma/diffuse large B-cell lymphoma, a clinico-pathological series of 25 cases. Br J Haematol. (2020) 189:244–56. doi: 10.1111/bjh.v189.2 32030731

[6] Di J Wei S Jackson A Munker R Kesler MV . Transition from Epstein-Barr virus (EBV)-positive rectal Hodgkin lymphoma to diffuse large B-cell lymphoma in the lung. Cureus. (2024). Available online at: https://www.cureus.com/articles/268383-transition-from-epstein-barr-virus-ebv-positive-rectal-hodgkin-lymphoma-to-diffuse-large-b-cell-lymphoma-in-the-lung. 10.7759/cureus.65013 PMC11333787 39165470

[7] Van Den Brand M Scheijen B Hess CJ Van Krieken JHJ Groenen PJTA . Pathways towards indolent B-cell lymphoma — Etiology and therapeutic strategies. Blood Rev. (2017) 31:426–35. doi: 10.1016/j.blre.2017.08.002 28802906

[8] Roark CL Ho BE Aubrey MT Anobile C Israeli S Phang TL . HLA homozygosity is associated with Non-Hodgkin lymphoma. Hum Immunol. (2022) 83:730–5. doi: 10.1016/j.humimm.2022.08.002 35953408

[9] Zhong C Gragert L Maiers M Hill BT Garcia-Gomez J Gendzekhadze K . The association between HLA and non-Hodgkin lymphoma subtypes, among a transplant-indicated population. Leuk Lymphoma. (2019) 60:2899–908. doi: 10.1080/10428194.2019.1617858 PMC6858537 31215275

[10] Cui Q Tan W Song B Peng RJ Wang L Dorajoo R . Genetic susceptibility of diffuse large B-cell lymphoma: a meta genome-wide association study in Asian population. Leukemia. (2024) 9(3):694–702. Available online at: https://www.nature.com/articles/s41375-024-02503-4. 10.1038/s41375-024-02503-4 39707004

[11] Diamanti I Fylaktou A Verrou E Vlachaki E Sinakos M Katodritou E . HLA variations in patients with diffuse large B-cell lymphoma and association with disease risk and prognosis: a case-control study. Front Genet. (2024) 15:1341822. doi: 10.3389/fgene.2024.1341822 38680423 PMC11045888

[12] Lu Y Abdou AM Cerhan JR Morton LM Severson RK Davis S . Human leukocyte antigen class I and II alleles and overall survival in diffuse large B-cell lymphoma and follicular lymphoma. Sci World J. (2011) 11:2062–70. doi: 10.1100/2011/373876 PMC3217596 22125456

[13] Fangazio M Ladewig E Gomez K Garcia-Ibanez L Kumar R Teruya-Feldstein J . Genetic mechanisms of HLA-I loss and immune escape in diffuse large B cell lymphoma. Proc Natl Acad Sci USA. (2021) 118:e2104504118. doi: 10.1073/pnas.2104504118 34050029 PMC8179151

[14] Alcoceba M Sebastián E Marín L Balanzategui A Sarasquete ME Chillón MC . HLA specificities are related to development and prognosis of diffuse large B-cell lymphoma. Blood. (2013) 122:1448–54. doi: 10.1182/blood-2013-02-483420 23843497

[15] Wang SS Abdou AM Morton LM Thomas R Cerhan JR Gao X . Human leukocyte antigen class I and II alleles in non-Hodgkin lymphoma etiology. Blood. (2010) 115:4820–3. doi: 10.1182/blood-2010-01-266775 PMC2890176 20385791

[16] Huang X Hepkema B Nolte I Kushekhar K Jongsma T Veenstra R . HLA-A*02:07 is a protective allele for EBV negative and a susceptibility allele for EBV positive classical Hodgkin lymphoma in China. PloS One. (2012) 7:e31865. doi: 10.1371/journal.pone.0031865 22355400 PMC3280205

[17] Fletcher LB Veenstra RN Loo EY Hwang AE Siddiqi IN Visser L . HLA expression and HLA type associations in relation to EBV status in Hispanic Hodgkin lymphoma patients. PloS One. (2017) 12:e0174457. doi: 10.1371/journal.pone.0174457 28334025 PMC5363938

[18] Wang Q-L Wang T-M Deng C-M Zhang W-L He Y-Q Xue W-Q . Association of HLA diversity with the risk of 25 cancers in the UK Biobank. eBioMedicine. (2023) 92:104588. doi: 10.1016/j.ebiom.2023.104588 37148584 PMC10189092

[19] Zakka LR Reche P Ahmed AR . Role of MHC Class II Genes in the pathogenesis of pemphigoid. Autoimmun Rev. (2011) 11:40–7. doi: 10.1016/j.autrev.2011.07.002 21782980

[20] Ekström Smedby K Vajdic CM Falster M Engels EA Martínez-Maza O Turner J . Autoimmune disorders and risk of non-Hodgkin lymphoma subtypes: a pooled analysis within the InterLymph Consortium. Blood. (2008) 111:4029–38. doi: 10.1182/blood-2007-10-119974 PMC2288717 18263783

[21] Stasiak M Zawadzka-Starczewska K Lewiński A . Significance of HLA haplotypes in two patients with subacute thyroiditis triggered by mRNA-based COVID-19 vaccine. Vaccines. (2022) 10:280. doi: 10.3390/vaccines10020280 35214738 PMC8879821

[22] Watad A Quaresma M Brown S Cohen Tervaert JW Rodríguez-Pint I Cervera R . Autoimmune/inflammatory syndrome induced by adjuvants (Shoenfeld’s syndrome) – An update. Lupus. (2017) 26:675–81. doi: 10.1177/0961203316686406 28059022

[23] Yu Y Shi X Wang X Zhang P Bai O Li Y . Second Malignant neoplasms in lymphomas, secondary lymphomas and lymphomas in metabolic disorders/diseases. Cell Biosci. (2022) 12:30. doi: 10.1186/s13578-022-00763-0 35279210 PMC8917635

[24] On behalf of the European Intergroup for Childhood Non-Hodgkin’s Lymphoma (EICNHL) and the International Berlin-Frankfurt-Münster (i-BFM) Study Group Attarbaschi A Carraro E Ronceray L Andrés M Barzilai-Birenboim S . Second Malignant neoplasms after treatment of non-Hodgkin’s lymphoma—a retrospective multinational study of 189 children and adolescents. Leukemia. (2021) 35:534–49. doi: 10.1038/s41375-020-0841-x 32393843

[25] Salvetti C Vitale C Griggio V Drandi D Jones R Bonello L . Case report: sequential development of three mature lymphoid neoplasms in a single patient: clonal relationship and molecular insights. Front Oncol. (2022) 12:917115. doi: 10.3389/fonc.2022.917115 35734588 PMC9207196

